# An evaluation of the osmole gap as a screening test for toxic alcohol poisoning

**DOI:** 10.1186/1471-227X-8-5

**Published:** 2008-04-28

**Authors:** Larry D Lynd, Kathryn J Richardson, Roy A Purssell, Riyad B Abu-Laban, Jeffery R Brubacher, Katherine J Lepik, Marco LA Sivilotti

**Affiliations:** 1 Faculty of Pharmaceutical Sciences, University of British Columbia, Vancouver, BC, Canada; 2 Centre for Health Evaluation and Outcomes Sciences, Providence Health Care, Vancouver, Canada; 3 Department of Emergency Medicine, Vancouver General Hospital, Vancouver, Canada; 4 British Columbia Drug and Poison Information Centre, Provincial Health Services Authority of BC, Vancouver, Canada; 5 Division of Emergency Medicine, Dept. of Surgery, Faculty of Medicine, University of British Columbia; Vancouver; Canada; 6 Centre for Clinical Epidemiology and Evaluation, Vancouver Coastal Health Research Institute, Vancouver, Canada; 7 Departments of Emergency Medicine and of Pharmacology & Toxicology, Queen's University, Kingston, Canada; and Ontario Poison Centre, Toronto, Canada

## Abstract

**Background:**

The osmole gap is used routinely as a screening test for the presence of exogenous osmotically active substances, such as the toxic alcohols ethylene glycol and methanol, particularly when the ability to measure serum concentrations of the substances is not available. The objectives of this study were: 1) to measure the diagnostic accuracy of the osmole gap for screening for ethylene glycol and methanol exposure, and 2) to identify whether a recently proposed modification of the ethanol coefficient affects the diagnostic accuracy.

**Methods:**

Electronic laboratory records from two tertiary-care hospitals were searched to identify all patients for whom a serum ethylene glycol and methanol measurement was ordered between January 1, 1996 and March 31, 2002. Cases were eligible for analysis if serum sodium, blood urea nitrogen, glucose, ethanol, ethylene glycol, methanol, and osmolality were measured simultaneously. Serum molarity was calculated using the Smithline and Gardner equation and ethanol coefficients of 1 and 1.25 mOsm/mM. The diagnostic accuracy of the osmole gap was evaluated for identifying patients with toxic alcohol levels above the recommended threshold for antidotal therapy and hemodialysis using receiver-operator characteristic curves, likelihood ratios, and positive and negative predictive values.

**Results:**

One hundred and thirty-one patients were included in the analysis, 20 of whom had ethylene glycol or methanol serum concentrations above the threshold for antidotal therapy. The use of an ethanol coefficient of 1.25 mOsm/mM yielded higher specificities and positive predictive values, without affecting sensitivity and negative predictive values. Employing an osmole gap threshold of 10 for the identification of patients requiring antidotal therapy resulted in a sensitivity of 0.9 and 0.85, and a specificity of 0.22 and 0. 5, with equations 1 and 2 respectively. The sensitivity increased to 1 for both equations for the identification of patients requiring dialysis.

**Conclusion:**

In this sample, an osmole gap threshold of 10 has a sensitivity and negative predictive value of 1 for identifying patients for whom hemodialysis is recommended, independent of the ethanol coefficient applied. In patients potentially requiring antidotal therapy, applying an ethanol coefficient of 1.25 resulted in a higher specificity and positive predictive value without compromising the sensitivity.

## Background

Serum osmolality can be measured directly (the 'measured osmolality') using osmometry, or estimated based on the direct measurement of the concentrations of the principle osmotically active substances (i.e. sodium, glucose, blood urea nitrogen, and ethanol) and then substituting these values into a formula to determine the 'calculated molarity'. The difference between the measured osmolality and the calculated molarity is referred to as the osmole gap [1,2]. The osmole gap is routinely used to screen patients for the presence of other exogenous osmotically active substances such as ethylene glycol and methanol, particularly when the ability to measure the serum concentrations of these substances is not available.

Screening and diagnostic tests are generally used to classify asymptomatic patients with respect to the likelihood of the presence of a disease [3]. Screening tests are ideally suited to detect diseases with a latent period between onset of disease (or time of exposure) and the development of overt symptoms, especially when the early diagnosis and initiation of therapy improves prognosis [4,5]. Toxic alcohol exposure meets these criteria given that serious toxicity is preventable with early diagnosis and initiation of antidotal therapy. The rapid and accurate diagnosis of toxic alcohol poisoning is therefore crucial to prevent serious adverse outcomes.

In a recent review of the medical literature we did not identify any well-designed studies of the osmole gap as a screening test for toxic alcohol exposure [1]. Numerous studies have either proposed a formula or formulae for estimating serum osmolality [6-14], evaluated the relationship between measured osmolality and calculated molarity in non-poisoned patient [6,7,10-12,14,15], or tested the ability of the osmole gap to predict serum ethanol concentrations in patients exposed only to ethanol [13,16-22]. While none of these studies provide evidence of the diagnostic performance of the osmole gap, they form the basis for the widespread use of the osmole gap as a screening test for toxic alcohol exposure.

To evaluate the osmole gap as a screening test, its performance must be compared to a gold standard diagnostic test (e.g. gas chromatography) in a sufficient number of patients at all levels of exposure with a specific definition of what constitutes a positive test (i.e. the diagnostic threshold of the osmole gap) [23]. We have not found any studies published to date that satisfy these criteria. Therefore, the objectives of this study were: 1) to measure the diagnostic accuracy of the osmole gap for screening for ethylene glycol and methanol exposure, and 2) to identify whether a recently proposed modification of the ethanol coefficient affects this diagnostic accuracy.

## Methods

### Setting

We conducted a retrospective analysis of laboratory records available from two tertiary care hospitals. Electronic laboratory records from both hospitals were searched to identify all patients with a serum ethylene glycol and methanol measurement recorded between January 1, 1996 and March 31, 2002. This study was approved by the institutional ethics review boards.

### Selection of Study Subjects

Cases were only eligible for inclusion in the analysis if serum sodium, blood urea nitrogen, glucose, ethanol, ethylene glycol, methanol, and serum osmolality measured using freezing point depression were measured on blood drawn at the same time. Cases were excluded if additional laboratory results indicated lipemia, ketosis, dysproteinemia, or hemolysis. Cases with a serum ethylene glycol and methanol level of 0 mmol/L and an arterial pH below 7.30 were deemed to have either a significant delay between exposure and clinical assessment, or another cause for the acidemia, and were also excluded from the analysis. In the event of multiple hospital visits only the first visit was included in the analysis.

### Methods of Measurement

In both hospitals, serum electrolytes, BUN, glucose and ethanol concentrations were determined using a high volume analyzer (Beckman CX7, Model 7566, Beckman Instruments, Inc. Fullerton, CA, USA) and serum osmolality was measured by freezing point depression (Advanced Micro Osmometer model 3300, Advanced Instruments Inc., Norwood, MA, USA). Serum concentrations of ethylene glycol and methanol were determined using gas chromatography (Hewlett Packard 5890A Gas Chromatograph, Hewlett Packard, Avondale, PA, USA), predefined as the gold standard for the diagnosis of toxic alcohol exposure.

### Data Analysis

The calculated molarity was obtained using the equation proposed by Smithline and Gardner which was deemed to be the equation applied most frequently by clinicians [9,24,25]. The contribution of ethanol to the osmolarity of serum was incorporated using two different coefficients: 1 mOsm/mM (equation 1), as has been standard practice [13], and 1.25 mOsm/mM (equation 2) as proposed by Purssell et al. [18,26]. The osmole gap was then calculated for each patient using both equations at the first instance when all required laboratory parameters were measured simultaneously, provided that this occurred within 24 hours of the first recorded laboratory measurement.

For the purposes of this analysis, a "positive exposure" was defined *a priori *as any serum ethylene glycol or methanol concentration above the recommended treatment thresholds. Specifically, we evaluated the ability of the osmole gap to identify patients with a serum ethylene glycol or methanol concentration above which antidotal therapy (3 mmol/L and 6 mmol/L, respectively) or hemodialysis (8 mmol/L and 15 mmol/L, respectively) is recommended [27,28].

The diagnostic accuracy of each equation was determined for all possible cut-offs of the osmole gap using Receiver-Operator Characteristics (ROC) curves. Because an osmole gap of 10 is the most common clinically applied cut-off for the diagnosis of potential toxic alcohol poisoning [27,29], we calculated the sensitivity, specificity, and positive and negative likelihood ratios of the test at this threshold. If the sensitivity was < 1.0, the hospital chart of every patient falsely classified as unexposed using this cut-off was reviewed to characterize their clinical course and outcome. Based on the conclusions of a study by Aabakken et al. [15], we also performed a secondary exploratory analysis of the diagnostic performance of an osmole gap of 20.

Receiver-operator characteristics curves were plotted using both equations for each treatment threshold. The area under the curve (AUC), or diagnostic index, was then calculated for each ROC curve. Non-parametric statistical analyses were used to determine whether the AUC for each ROC curve differed significantly from 0.5. In order to identify the equation with the best diagnostic performance, the difference in the diagnostic index of each equation was compared, using a non-parametric method that accounts for correlation within individuals [30]. The positive predictive value (PPV) and negative predictive value (NPV) of each equation was also calculated and plotted against all possible osmole gap cut-offs. All analyses were performed using SPSS v 12.0. (SPSS Inc., Chicago, IL, USA. 2003) and SAS v.8.02 (SAS Institute, Cary, NC, USA. 1999).

## Results

### Characteristics of Study Subjects

**Figure 1:**
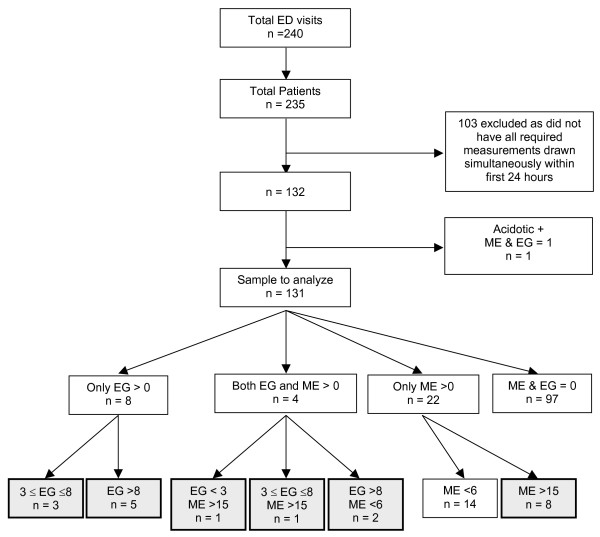
**Flow diagram outlining the derivation of the final study sample. **Shaded boxes represent patients considered to be 'exposed' (i.e. ethylene glycol or methanol serum concentrations exceeding the threshold for antidotal therapy). Legend: All concentrations expressed as mmol/l. EG = ethylene glycol ME = methanol

We identified 235 patients with 240 hospital visits during which serum ethylene glycol and methanol levels were measured by gas chromatography within 24 hours of their first laboratory results (Figure 1). Five patients had two separate hospital visits with toxic alcohols measurements, so only the first visit was included in the analysis. One hundred and three patients were excluded because they did not have all required measurements performed on serum drawn at the same time; 51 had toxic alcohol levels requisitioned but not measured suggesting that they were deemed to not be required (i.e. very low pre-test probability of exposure) and 37 had undetectable ethylene glycol and methanol concentrations. The remaining 15 patients had detectable toxic alcohol serum concentrations, nine of which exceeded the threshold for antidotal therapy. An osmole gap prior to the measurement of the toxic alcohol concentration could only be calculated for two of these patients, both of which exceeded 10 using both equation 1 and 2. One additional patient was excluded due to the presence of acidosis and no detectable toxic alcohol.

The final study population therefore included 131 patients, 20 of whom were deemed 'exposed' (i.e. had concentrations above the threshold for antidotal therapy) to either ethylene glycol (n = 10), methanol (n = 9), or both (n = 1). The only patient positive for exposure to both ethylene glycol and methanol had serum concentrations of 3 mmol/L and 122 mmol/L, respectively. Seventeen patients had serum ethylene glycol (n = 7) and methanol (n = 10) concentrations above the threshold for hemodialysis.

All detectable serum ethylene glycol concentrations exceeded the threshold for treatment (i.e. 3 mmol/L), ranging from 3 to 68.7 mmol/L (median 6, IQR 19.3). Serum methanol concentrations in patients considered exposed to methanol ranged from 16 to 202 mmol/L (median 56.4, IQR 100.3). Fourteen additional patients had methanol levels below the threshold for antidotal therapy, 11 of which had an osmole gap > 10 derived using equation 1 (mean OG 19.3, SD 15.4) versus only eight using equation 2 (mean OG 13.1, SD 18.1). These patients were therefore considered false-positive exposures. Seventy-six patients (58%) had positive serum ethanol levels (mean 44.2 mmol/L; range 0.3 to 135 mmol/L), seven of which exceeded 100 mmol/L.

### Main Results

**Figure 2:**
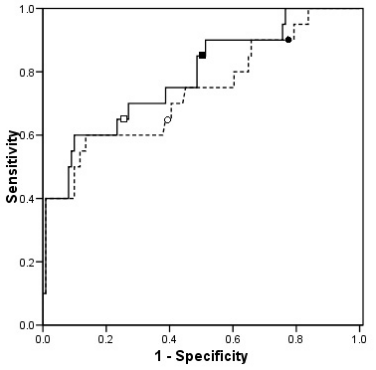
Receiver Operator Characteristics curves for the two equations when used to identify patients with serum concentrations of ethylene glycol and methanol that exceed the threshold at which antidotal therapy is recommended - - - - osmole gap derived using equation 1 (ethanol coefficient of 1) ------ osmole gap derived using equation 2 (ethanol coefficient of 1.25). The points delineated by ● and ■ indicate the cut-off of 10 derived using equations 1 and 2, respectively. The points delineated by ○ and □ indicate the cut-off of 20 derived using equations 1 and 2, respectively.

The results of the primary analyses of the osmole gap for identifying patients with toxic alcohol concentrations above the threshold for antidotal therapy are illustrated in Figure 2, and the associated diagnostic indices are presented in Table 2. Both equations resulted in an AUC that differed significantly from 0.5 (p < 0.001) suggesting that the osmole gap provides at least some discriminatory diagnostic information. Although equation 2 resulted in a higher diagnostic index relative to equation 1 (0.785 versus 0.736, respectively), this difference was not statistically significant (two sided p = 0.06).

Although the ROC curves and diagnostic indices associated with the two equations were similar, there were differences in their sensitivities and specificities at a given osmole gap (Figure 2). Applying an osmole gap cut-off of 10 for the identification of patients requiring antidotal therapy resulted in a sensitivity of 0.90 (95% CI, 0.68 – 0.99) with a corresponding specificity of only 0.22 (95% CI, 0.14 – 0.30) (87/111 false positive diagnoses) with equation 1. Equation 2 resulted in a slightly lower sensitivity of 0.85 (95% CI, 0.62 – 0.97) as a result of 1 additional false-negative case), but a higher specificity of 0.50 (95% CI, 0.40 – 0.59). If applied clinically, the higher specificity of equation 2 would have resulted in 31 fewer patients receiving a false-positive diagnoses, all of which had serum ethanol concentrations >0 mmol/L. Equation 2 also produced more favourable positive (LR+) and negative (LR-) likelihood ratios of 1.68 (95% CI, 1.29 – 2.16) and 0.30 (95% CI, 0.10 – 0.72), respectively, versus 1.15 (95% CI, 0.96 – 1.36) and 0.46 (95% CI, 0.11 – 1.30) respectively, for equation 1. Exploratory analysis of an osmole gap cut-off of 20 proposed by Aabakken et al. [15] resulted in a lower sensitivity with both equations 1 and 2 (0.65; 95% CI 0.41 – 0.85) and correspondingly more false-negative diagnoses (n = 7), but a higher specificity (0.60; 95% CI 0.51 – 0.69, and 0.75; 95% CI 0.66 – 0.83 with equations 1 and 2, respectively).

**Figure 3:**
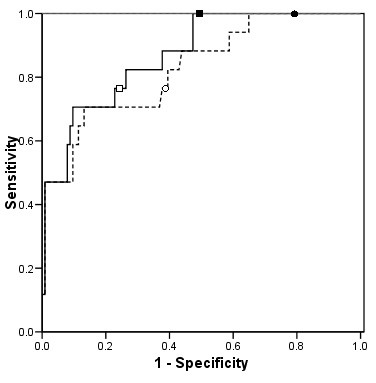
Receiver Operator Characteristics curves for the two equations when used to identify patients with serum concentrations of ethylene glycol and methanol that exceed the threshold at which hemodialysis is recommended - - - - osmole gap derived using equation 1 (ethanol coefficient of 1) ----- osmole gap derived using equation 2 (ethanol coefficient of 1.25). The points delineated by ● and ■ indicate an osmole gap cut-off of 10 derived using equations 1 and 2, respectively. The points delineated by ○ and □ indicate an osmole gap cut-off of 20 derived using equations 1 and 2, respectively.

Consistent with the results for the threshold for antidotal therapy, equation 2 resulted in a higher diagnostic index relative to equation 1 for the identification of patients requiring hemodialysis (0.827 versus 0.870, respectively); however, this difference was not statistically significant (p = 0.056) (Table 2). Figure 3 illustrates the ROC curves for the identification of patients with serum ethylene glycol or methanol concentrations above the threshold for hemodialysis. A cut-off of 10 resulted in a sensitivity of 1.0 (95% CI, 0.80 – 1.00) for both equations, but equation 2 was more specific (0.51; 95% CI, 0.41 – 0.60 versus 0.23; 95% CI, 0.15 – 0.32). The positive and negative likelihood ratios for equation 2 at a cut-off of 10 were 2.04 (95% CI, 1.68 – 2.44) and 0, respectively. Applying an osmole gap of 20 resulted in a decrease in sensitivity with both equations (0.76; 95% CI 0.50 – 0.93) and a slight improvement in specificity (0.61; 95% CI 0.52 – 0.70, and 0.76; 95% CI 0.67 – 0.83 with equation 1 and 2, respectively).

**Figure 4:**
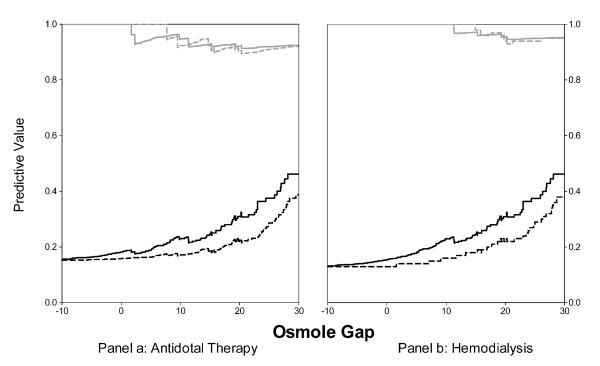
The Positive Predictive Value and Negative Predictive Value of osmole gap values ranging between -10 and 30 to identify toxic alcohol concentrations exceeding the antidotal therapy (Panel a) and hemodialysis (Panel b) thresholds Black lines represent the positive predictive value. Grey lines represent the negative predictive value. - - - - osmole gap derived using equation 1 (ethanol coefficient of 1) ----- osmole gap derived using equation 2 (ethanol coefficient of 1.25)

The PPV and NPV of all osmole gap cut-offs derived using both equations are illustrated in Figure 4. At an osmole gap cut-off of 10, the NPV of equations 1 and 2 were 0.92 (95% CI, 0.75 – 0.99) and 0.95 (95% CI, 0.86 – 0.99), respectively, for the identification of patients with levels above the threshold for antidotal therapy (Panel a). For the identification of patients with levels above the hemodialysis threshold (Panel b), both equations had a NPV of 1.0. The PPV of equation 2 was also consistently higher, independent of the osmole gap cut-off.

Because a false negative diagnosis of toxic alcohol exposure may result in potentially life-threatening or long-term sequelae, we reviewed the health records of the three patients with an osmole gap < 10 derived using either equation despite a serum ethylene glycol concentration above the threshold for antidotal therapy.

### Clinical Summary of False-Negative Cases (osmole gap threshold 10)

#### Case 1

A 55 year old male was admitted to an emergency department at least seven hours post-exposure to ethylene glycol, obtunded and requiring intubation. Initial laboratory results revealed an anion gap metabolic acidosis (HCO_3_^- ^3 mEq/L, anion gap 31.5) and an osmole gap of 143 (measured osmolality 446 mOsm/kg; calculated molarity 302.1 mOsm/L derived using equation 2). The patient was treated with intravenous ethanol, thiamine and folate prior to transfer to a tertiary care facility without confirmatory methanol or ethylene glycol concentrations. However, when all required laboratory parameters were measured simultaneously, he had an osmole gap of 7.8 (derived using equation 2) and an ethylene glycol level of 6 mmol/L. The patient underwent 13.5 hours of dialysis and developed acute renal failure with a peak creatinine of 1,410 mmol/L nine days post-exposure, and was discharged from hospital 19 days post-exposure with a serum creatinine of 613 mmol/L.

#### Case 2

A 51 year old female with a history of alcoholism, hepatitis C, hypertension, non-insulin dependent diabetes mellitus, and intravenous drug use was admitted to the emergency department following a near drowning episode. Although she was initially unresponsive and cyanotic, upon arrival at the emergency department she was alert but confused. Initial lab results revealed an anion gap metabolic acidosis (pH 7.1, HCO_3_^- ^11 mEq/L, anion gap 27) with an osmole gap of 19.2 calculated using equation 2. Three and one-half hours after admission (the time point used in this analysis), her serum ethylene glycol, methanol, and ethanol concentrations were 5 mmol/L, 0 mmol/L, 54 mmol/L, respectively, with a corresponding osmole gap of 1.7 (calculated using equation 2). She did not receive any further antidotal therapy or hemodialysis and was discharged following two days of hospitalization without any adverse sequelae.

#### Case 3

A 50 year old developmentally delayed male with a prior history of ethylene glycol ingestion presented to the emergency department approximately 7.5 hours after an intentional ingestion of approximately 400 ml of ethylene glycol. Twenty minutes after arrival, laboratory results revealed an anion gap metabolic acidosis (arterial pH 7.27, HCO_3_^- ^9.4 mEq/L, anion gap 18), an osmole gap of 9.8, and an ethylene glycol concentration of 5 mmol/L; ethanol was undetectable. An ethanol infusion was initiated within one hour of presentation followed four hours later by five hours and forty minutes of hemodialysis. His serum creatinine peaked five days post-exposure at 459 mmol/L, following which he recovered completely.

## Discussion

This is the first study to evaluate the osmole gap as a screening test for toxic alcohol exposure that conforms to the STARD criteria for reporting studies of diagnostic accuracy [31]. In this sample, an osmole gap cut-off of 10 derived using the Smithline and Gardner equation with ethanol coefficients of 1 and 1.25 resulted in a relatively high sensitivity (> 0.85) but a low specificity (< 0.50) and high NPVs for the identification of patients for whom antidotal therapy for toxic alcohol poisoning would be indicated. These results therefore indicate that although this is not an ideal screening test, the osmole gap does provide additional diagnostic information. Specifically, a NPV of 0.95 for an osmole gap < 10 indicates a very high probability that if a toxic alcohol has been ingested, the serum level is below the threshold for antidotal therapy. This analysis does not suggest that the osmole gap should be used in isolation to provide the basis for discharging a patient without further clinical investigation or evaluation. However, it does indicate that the osmole gap does provide some additional diagnostic and prognostic information in terms of the probability that a patient will need antidotal therapy or hemodialysis. Using other clinical information and laboratory data in conjunction with the osmole gap will increase the accuracy of the diagnosis.

Using equation 2 to account for the supramolar contribution of ethanol resulted in an increase in the specificity of the osmole gap without a significant reduction in sensitivity. Although a screening test with a sensitivity of 1.0 is most desirable in this clinical situation, achieving this would result in a low specificity and a corresponding high false-positive rate which could result in the unnecessary initiation of treatment in these patients. Although antidotal treatment with either intravenous ethanol or fomepizole is relatively benign, the cost-effectiveness of any screening test must incorporate all costs and potential risks of misdiagnosis and inappropriate initiation of treatment which might include unnecessary hospitalization, treatment, or transfer by air or road ambulance.

Of the three exposed patients who may have been falsely diagnosed as unexposed using an osmole gap cut-off of 10, two had an elevated osmole gap upon presentation to the emergency department and would likely have been correctly diagnosed as exposed. The third patient had a compelling history of significant ethylene glycol ingestion, an osmole gap of 9.8, and an anion gap metabolic acidosis when they arrived at the emergency room and would also likely have been correctly diagnosed based on other available information.

A survey of studies of diagnostic accuracy published in four major journals concluded that the methodologies employed were generally inadequate to answer the questions posed [32]. The results of that survey lead to the development of the Standards for Reporting Diagnostic Accuracy (STARD) statement which consists of 25 criteria that should be adhered to when evaluating the diagnostic accuracy of a test [31,33]. This is the first formal evaluation of the diagnostic accuracy of the osmole gap that: i) includes subjects with all levels of exposure to ethylene glycol or methanol; ii) evaluates the osmole gap compared to the gold standard; and iii) conforms to essentially all other STARD criteria.

One of the necessary components of any screening test evaluation is a clear and valid definition of what constitutes a positive test [23]. Although Aabakken et al. proposed a threshold of 20 using a different equation to calculate serum osmolarity, this was based on 177 consecutive patients admitted to an ED (mean age 65 years) without any exposure to ethylene glycol or methanol [15]. Given that the normal range for the osmole gap may be higher in patients older than 60 years of age and that the osmole gap is generally used in conjunction with other diagnostic information, we felt that it is unlikely that this threshold is applicable to the diagnosis of toxic alcohol poisoning [34]. In support of this, although applying an osmole gap cut-off of 20 in our sample resulted in higher specificity relative to an osmole gap of 10, it had a lower sensitivity that corresponded to six additional false-negative diagnoses [15]. This reduction in sensitivity is concerning in this clinical scenario, given the potential serious and fatal ramifications of a false-negative diagnosis.

The diagnostic performance of the osmole gap is related to both the cut-off of the test and serum level being detected. Because we were unable to find any other empiric evaluation of the most appropriate osmole gap cut-off for the diagnosis of toxic alcohol poisoning, we elected to evaluate all possible cut-offs using ROC curves, and then specifically evaluate the clinically accepted osmole gap threshold of 10. Additionally, there is limited evidence supporting the current recommended thresholds of ethylene glycol and methanol concentrations for initiating antidotal therapy and hemodialysis that we used in this analysis. If these thresholds are too conservative as has been suggested, more liberal treatment thresholds would improve the diagnostic performance of the osmole gap [35].

Because the inclusion criteria applied in this study required that both ethylene glycol and methanol be measured by gas chromatography, some patients may have had a toxic alcohol level above the threshold for antidotal therapy that was not measured. The diagnostic performance of the osmole gap may therefore be poorer if applied to all patients with any suspicion of exposure, including cases where exposure may have been potentially ruled out, albeit erroneously, before specific serum concentration measurements are performed.

This highlights the importance of interpreting the result of the osmole gap as it relates to other available information to estimate the likelihood of a toxic alcohol exposure. Information pertaining to the ingestion history, clinical signs and symptoms (e.g. visual disturbances) and other laboratory data including pH, HCO_3_^-^, anion gap and urinalysis should all be considered concurrently [36]. However, this study only evaluated the diagnostic accuracy of the osmole gap alone measured within 24 hours of the first recorded laboratory measurement. Consequently, these results are likely an underestimate of the overall clinical utility of the osmole gap when evaluated in conjunction with other diagnostic information as early as possible following exposure.

This study has three primary limitations. First, it is limited by the use of retrospective data and the requirement that all laboratory tests be performed on serum obtained simultaneously; this resulted in the exclusion of 103 subjects. However, to avoid making clinical assumptions using only laboratory data, we pre-specified that all required laboratory parameters had to be measured simultaneously. Because these measurements were not necessarily performed on the first blood sample obtained upon presentation to the emergency room, we restricted this analysis to only laboratory data collected within the first 24 hours of the first available blood sample. The rationale for this restriction was to mimic admission results as closely as possible within the constraints of a retrospective study. Realizing the critical importance of the simultaneous evaluation of all laboratory parameters immediately upon admission, and the time of the ingestion when interpreting the osmole gap, a prospective study is required to specifically address this methodological issue.

The second limitation of the study is the potential for referral or ascertainment bias. This sample only included patients who had serum ethylene glycol and methanol levels measured, which might suggest a high pre-test probability of exposure. However, ethylene glycol or methanol was only detectable in 34/131 (26%) patients. This incidence of detectable toxic alcohol concentrations suggests that the referral bias was not extreme and that the pre-test probability of exposure in this sample of patients was likely only moderate.

Finally, there is the potential for work-up bias. If an osmole gap above a specific cut-off is used as a prerequisite to measuring toxic alcohols using gas chromatography, this would tend to overstate the sensitivity of the test. We cannot exclude this possibility, especially in patients with toxic alcohol concentrations just above the threshold for antidotal therapy. Although it is possible that some of the 51 patients who had ethylene glycol and methanol concentrations requisitioned but not measured may have had elevated toxic alcohol concentrations despite an osmole gap below 10, we believe that workup bias was not significant in this dataset for several reasons. First, gas chromatography was performed in 25 patients despite an osmole gap below 10 calculated using equation 1, the most commonly applied equation in clinical practice. Second, we are not aware of any patient in either institution during the study period with an osmole gap less than 10 that subsequently developed significant toxicity. And finally, most of the cases with a detectable methanol concentration below the threshold for antidotal therapy had an osmole gap > 10.

## Conclusion

Toxic alcohol exposure is a clinical emergency requiring rapid evaluation and initiation of treatment to prevent serious morbidity and mortality. Unfortunately, making a definitive diagnosis is often difficult given that the gold standard test (i.e. gas chromatography) is not available at most hospitals. As a result, the inexpensive and widely available osmole gap remains part of the diagnostic strategy for most emergency room physicians, despite two important limitations [13,37]. First, the osmole gap lacks specificity, given that it is also elevated in other clinical situations, e.g. diabetic ketoacidosis, circulatory shock, and alcoholic acidosis [1]. Second, its wide normal range renders it insensitive to small but potentially toxic concentrations of ethylene glycol in particular, but also methanol [38]. Despite these limitations, until now there has not been a rigorous, methodologically sound evaluation of diagnostic accuracy of this test for the diagnosis of toxic alcohol exposure.

The results of this analysis indicate that the osmole gap does provide additional diagnostic information when applied as a screening test for toxic alcohol poisoning, and its' diagnostic accuracy improves when the supramolar contribution of ethanol to serum molarity is taken into account. However, these results do not support the use of the osmole gap in isolation, and further support the conclusions of Krahn and Khajuria who suggest that these calculations are only effective if they are validated on appropriate reference populations, and if strict quality control procedures are followed [39]. Therefore, a multi-centre prospective evaluation of the osmole gap is required to evaluate the overall clinical utility of the test, taking into account the time since ingestion, other laboratory results, all available clinical diagnostic information collected immediately upon admission, and institutional differences.

## Competing interests

The authors declare that they have no competing interests.

## Authors' contributions

LDL was involved in the study concept and design, analysis and interpretation of the data, drafting and final revisions of the manuscript, and oversaw the study. KJR undertook the statistical analysis and participated in the drafting of the manuscript. RAP, RBAL and JRB all contributed to the study concept and design, data interpretation, and critical revision of the manuscript. RBAL was also involved in the acquisition of the data. KJL contributed to the study design and concept, critical evaluation of the manuscript, and data acquisition, and MLAS participated in the acquisition of the data, analysis and interpretation of the data, and critical revision of the manuscript. All authors have read and approved the final manuscript.

**Table 1:** Equations used to calculate the serum molarity prior to the calculation of the osmole gap.

**Equation Number**	**Equation**
1	Osm_c _= 2 * Na (mEq/L) + BUN (mmol/L) + glucose (mmol/L) + ethanol (mmol/L)
2	Osm_c _= 2 * Na (mEq/L) + BUN (mmol/L) + glucose (mmol/L) + 1.25 * ethanol (mmol/L)

Equation 2 in effect inflates the ethanol concentration by 25% prior to calculating the osmole gap, as proposed by Purssell et al.[18]

Osm_c _= calculated molarity; Na = sodium; BUN = blood urea nitrogen

To convert from SI units, use the following corrections: BUN/2.8 mg/dl, glucose/18.1 mg/dl, ethanol/0.217 mg/dl

**Table 2:** Area under the curve (diagnostic index) for osmole gap to identify toxic alcohol exposure.

**Exposure Threshold**	**AUC (95% CI)**	**p value**	**AUC_2 _- AUC_1 _(95% CI)**	**p value**
**ANTIDOTAL THERAPY**				
Equation 1	0.736 (0.599, 0.873)	< 0.001	0.049 (-0.001, 0.099)	0.057
Equation 2	0.785 (0.665, 0.905)	< 0.001		
**HEMODIALYSIS**				
Equation 1	0.827 (0.715, 0.939)	< 0.001	0.043 (-0.001, 0.086)	0.056
Equation 2	0.870 (0.784, 0.956)	< 0.001		

AUC = area under the curve; SE = standard error; CI = confidence interval

## Pre-publication history

The pre-publication history for this paper can be accessed here:


